# Characterizing Aging in the Human Brainstem Using Quantitative Multimodal MRI Analysis

**DOI:** 10.3389/fnhum.2013.00462

**Published:** 2013-08-20

**Authors:** Christian Lambert, Rumana Chowdhury, Thomas H. B. FitzGerald, Stephen M. Fleming, Antoine Lutti, Chloe Hutton, Bogdan Draganski, Richard Frackowiak, John Ashburner

**Affiliations:** ^1^Clinical Neuroscience, St George’s University of London, London, UK; ^2^Wellcome Trust Centre for Neuroimaging, UCL Institute of Neurology, University College London, London, UK; ^3^Center for Neural Science, New York University, New York, NY, USA; ^4^Department of Experimental Psychology, University of Oxford, Oxford, UK; ^5^LREN, Département des Neurosciences Cliniques, CHUV, Université de Lausanne, Lausanne, Switzerland

**Keywords:** brainstem, quantitative MRI, tensor-based morphometry, voxel-based quantification, aging

## Abstract

Aging is ubiquitous to the human condition. The MRI correlates of healthy aging have been extensively investigated using a range of modalities, including volumetric MRI, quantitative MRI (qMRI), and diffusion tensor imaging. Despite this, the reported brainstem related changes remain sparse. This is, in part, due to the technical and methodological limitations in quantitatively assessing and statistically analyzing this region. By utilizing a new method of brainstem segmentation, a large cohort of 100 healthy adults were assessed in this study for the effects of aging within the human brainstem *in vivo*. Using qMRI, tensor-based morphometry (TBM), and voxel-based quantification (VBQ), the volumetric and quantitative changes across healthy adults between 19 and 75 years were characterized. In addition to the increased R2* in substantia nigra corresponding to increasing iron deposition with age, several novel findings were reported in the current study. These include selective volumetric loss of the brachium conjunctivum, with a corresponding decrease in magnetization transfer and increase in proton density (PD), accounting for the previously described “*midbrain shrinkage*.” Additionally, we found increases in R1 and PD in several pontine and medullary structures. We consider these changes in the context of well-characterized, functional age-related changes, and propose potential biophysical mechanisms. This study provides detailed quantitative analysis of the internal architecture of the brainstem and provides a baseline for further studies of neurodegenerative diseases that are characterized by early, pre-clinical involvement of the brainstem, such as Parkinson’s and Alzheimer’s diseases.

## Introduction

Aging is ubiquitous to the human condition. Previously, the cortical and subcortical correlates of normal aging have been extensively investigated using several modalities including volumetric MRI (Jernigan et al., 2001; Walhovd et al., 2005, 2011), quantitative MRI (qMRI) (Armstrong et al., 2004; Draganski et al., 2011; Bilgic et al., 2012), cortical thickness measures (Thambisetty et al., 2010), and diffusion tensor imaging (DTI) (Pfefferbaum et al., 2010; Vaillancourt et al., 2012). These studies have established that the normal aging process is associated with distinctive morphological changes including volumetric loss of the cortex, predominantly in the prefrontal, parietal, and temporal regions in addition to the amygdala, hippocampus, striatum, and cerebellum (Woodruff-Pak et al., 2010; Draganski et al., 2011; Walhovd et al., 2011). Corresponding with this, there is a reduction in cortical white-matter myelination, reflected by falling magnetization transfer (MT) values, and increasing iron deposition particularly in basal ganglia structures, notably the substantia nigra, as reflected by increasing R2* values (Pfefferbaum et al., 2009, 2010; Haacke et al., 2010; Draganski et al., 2011; Bilgic et al., 2012). Despite these widespread effects, reported brainstem related changes remain sparse due to technical limitations of imaging, segmenting, and statistically analyzing data from this region (Luft et al., 1999; Raz et al., 2001; Lee et al., 2009). Aging is known to negatively impact on several brainstem-mediated functions, for example the sleep-wake cycle (Hut and Van der Zee, 2011), sympathetic outflow (Samuels and Szabadi, 2008), vestibular-ocular reflexes (Baloh et al., 1993), and cardiovascular reflexes (Vita et al., 1986). Post-mortem reports indicate that changes within the brainstem sub-nuclei do take place during aging (Alvarez et al., 2000; Samuels and Szabadi, 2008), some of which may be early precursors for subclinical neurodegenerative disease (Tsopelas et al., 2011).

Quantitative MRI produces quantitative MR parameters that can be used as biomarkers of tissue microstructure (Tofts, 2003; Draganski et al., 2011). Examples of these parameters include MT, Proton Density (PD), R2*, and R1. MT emerges from hydrogen in motionally restricted macromolecules and more directly relates to macromolecular content. Biologically, it is a reflection of the quantity of myelin within a voxel (Helms et al., 2008b). It is important to note that in the literature MT contrast is often reported as “MT ratio” (Dousset et al., 1992), a parameter that shows residual T1 dependence. However, by using semi-quantitative parameter, the MT saturation, MT and T1 effect can be separated (Helms et al., 2008b). PD refers to the concentration of MRI-visible water (Tofts, 2003). R2* (=1/T2*) is the relaxation rate of the transverse magnetization, and is linearly correlated with tissue iron concentration (Yao et al., 2009). Finally R1 (=1/T1) is the longitudinal relaxation rate, and arises from a mix of water content, iron, and tissue macromolecule fraction (Rooney et al., 2007). Increases in iron (Rooney et al., 2007), decreases in PD (Gelman et al., 2001), and increases in lipid content (Stanisz et al., 2005) all cause an increase in the measured R1 signal.

The aim of this cross-sectional study was to characterize volumetric and tissue parameter changes associated with aging within the human brainstem *in vivo* using qMRI, tensor-based morphometry (TBM), and voxel-based quantification (VBQ). This was motivated by the observation that certain neurodegenerative diseases, such as Parkinson’s and Alzheimer’s diseases, are characterized by early pre-clinical involvement of the brainstem (Simic et al., 2009; Hawkes et al., 2010). In order to further study these effects, changes associated with the normal aging process must first be better characterized.

## Materials and Methods

### Subjects

Multiparametric maps were acquired from previous studies (FitzGerald et al., 2012; Lambert et al., 2012; Chowdhury et al., 2013). In total, imaging data for 100 healthy adults (47 males, aged 40.3 ± 21.2 years; 53 females, aged 48.2 ± 22.7 years; age ranged 19–75 years) who had MRI scanning at the Wellcome Trust Centre for Neuroimaging was used. Due to the selection criteria, these predominately fell into a bimodal distribution (as is shown Figure 5), with a younger cohort less than 60 years [*n* = 58, mean age 25.8 years (SD 7.6 years)] and an older cohort above 60 years [*n* = 42, mean age 69.1 years (SD 3.5 years)]. Subjects above 60 years had a normal neurological examination performed by a physician (Rumana Chowdhury), MMSE score>28 and a normal performance (within 1.5 SD of age-related norm) on a range of neuropsychological tests. Involvement of human volunteers was approved by the local ethics committee, and each subject provided written informed consent prior to MRI examination.

### Image acquisition

MR imaging was performed on a 3T whole-body MRI system (Magnetom TIM Trio, Siemens Healthcare, Erlangen) operated with a whole-body transmit radio-frequency (RF) coil and a 32-channel RF receive coil. MR data of 21 subjects was acquired on a second identical MRI system located within the same department. Each participant underwent a multiparametric mapping (MPM) scanning protocol for quantitative mapping of multiple MR parameters. MT, R1 (=1/T1), and PD weighted images were acquired using 3D multi-echo FLASH (fast low-angle shot) acquisitions (Helms et al., 2008a,b). Full imaging parameters are summarized in Table 1. The image resolution was 1 mm isotropic. The total acquisition time was 19 min. For each subject quantitative MT, R1, PD, and R2* (=1/T2*) maps were extracted from the acquired images using in-house MATLAB program. An additional dataset was acquired on each subject for mapping of the RF transmit field B1+ over the brain (4 mm isotropic resolution, acquisition time 3 min) using the 3D EPI SE/STE method described in (Lutti et al., 2010, 2012). A B0-field map was also acquired to correct for the distortions of the EPI images acquired for the B1+ mapping acquisition (acquisition time 2 min). The resulting B1+ maps were used to correct the MPM maps for RF transmit field inhomogeneity effects (Helms et al., 2008a). The PD maps were corrected for the spatially varying sensitivity profile of the receive coil using the UNICORT algorithm (Weiskopf et al., 2011). The resulting “flattened” signal amplitude maps were converted into PD maps by scaling the voxels by the expected average PD of white-matter [69% (Tofts, 2003)].

**Table 1:** **Imaging parameters**.

	Image type	Slice No	FOV (mm^2^)	Acquisition matrix (voxels)	TR (ms)	TE (ms)	Flip angle	Echo No.	Notes
Multispectral	MTw	176	240 × 256	240 × 256	23.7	[2.2:2.5:14.7]	6	6	Resolution = 1 mm
sequence	T1w	176	240 × 256	240 × 256	23.7	[2.2:2.5:14.7]	20	8	Parallel imaging (GRAPPA)
	PDw	176	240 × 256	240 × 256	18.7	[2.2:2.5:19.7]	6	6	along phase encoding direction
	B1-Map	48	192 × 256	48 × 64	500	(SE:37.06; STE:68.26)	SE:[230:−10:130]	2	Griswold et al. (2002)
	Fieldmap	64	192 × 192	64 × 64	1020	10; 12.46	90	2	Griswold et al. (2002) partition partial Fourier (6/8). Bandwidth = 425Hz/pixel

### Pre-processing

The MT maps were initially segmented into gray, white, and CSF tissue classes whilst maintaining the native resolution (1 mm) by using the unified segmentation within SPM8 (http://www.fil.ion.ucl.ac.uk/spm/) (Ashburner and Friston, 2005). For each individual, the total intracranial volume (TIV) was calculated by summing the gray matter, white-matter, and CSF segmentations at a threshold of 0.1. The segmentations were then registered diffeomorphically to a common group-average 1 mm isotropic template (using the “*Shoot toolbox*” of SPM, Ashburner and Friston, 2011) to produce deformation fields that were subsequently used in the segmentation step to warp the brainstem tissue priors to individual subject space.

**Figure 1:**
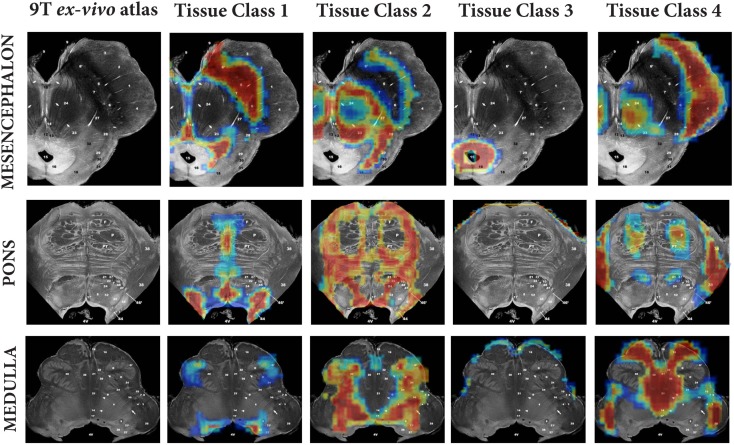
**Comparison of brainstem tissue classes against three corresponding *ex vivo* brainstem sections from “MR microscopy at 9.4T” (taken from Duvernoy’s Atlas of the Human Brain Stem and Cerebellum with permission)**. Figure reproduced from Lambert et al. (2013) with permission from Elsevier.

A method for generating brainstem specific tissue probability maps (TPMs) and subsequent segmentation had been previously developed (Lambert et al., 2013). In brief, the brainstem TPMs were defined using a modified multivariate Mixture of Gaussians (mmMoG) to generate spatial TPMs from 0.8 mm isotropic MT and PD maps, which were masked to include the brainstem from the origin of the cerebral aqueduct to the level of the foramen magnum. Four tissue classes were identified, three gray matter and one white-matter. These were labeled for descriptive purposes according to the predominant tissue type present: tissue class one included the substantia nigra, locus coeruleus and raphe nuclei and hence was designated “*monoaminergic gray matter*,” though regions that contained dorsal cranial nerve nuclei were also included. Tissue class two consisted mainly of the nucleus reticularis throughout its length and the pontine nuclei, and hence was designated “*reticulated gray matter*.” Tissue class three was specific for the periaqueductal gray (PAG) and labeled as such. Tissue class four was the brainstem white-matter. Examples of these tissue classes projected onto the corresponding sections from Duvernoy’s 9.4T MRI brainstem atlas (Naidich and Duvernoy, 2009) have been provided in Figure 1 (reprinted from Lambert et al., 2013 with permission from Elsevier).

The previously calculated tissue priors for four brainstem and one non-brainstem tissue classes were aligned with the calculated group-average template. This was achieved by realigning a whole brain template in the same space as the TPMs with the corresponding new group-average template, re-slicing to achieve 1 mm isotropic voxel size with a 4th degree spline interpolation and ensuring each individual voxel probability value summed to one across all the maps.

**Figure 2:**
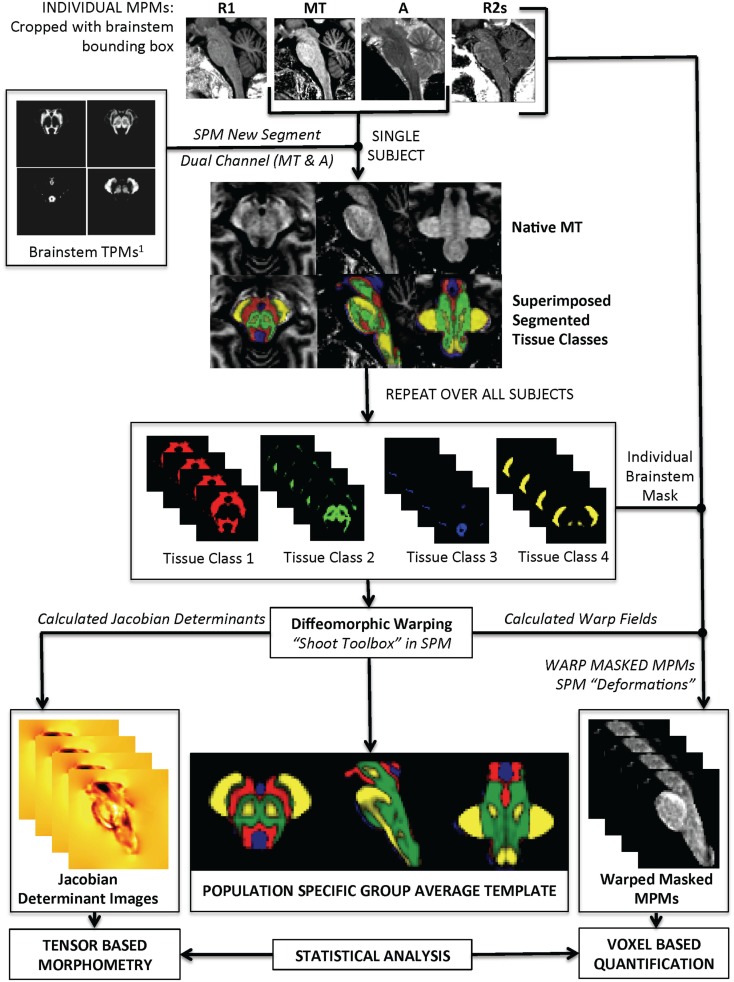
**Summary of segmentation pipeline**. This includes examples of the Multiparameter Maps (MPMs) as shown at the top of the image.

The actual brainstem segmentation step is summarized in Figure 2. The realigned probability maps were warped to individual subject space and used in SPM8 “*New Segment*” on individual MT and PD maps that had been cropped with a set-bounding box to include only the brainstem region. Specifically, five tissue classes were used; four within brainstem and one for everything else. Two Gaussians were used to model each tissue class except for the PAG matter (one Gaussian) and non-brainstem (eight Gaussians). Individual level segmentations were generated from the MT and PD images. All the images from each of the four tissue classes were then cropped using a common bounding box to increase computational speed and visually checked to ensure good quality. All of the brainstem tissue classes were then re-warped back to a group-average using a diffeomorphic warping algorithm (geodesic shooting) to allow re-estimation of the Jacobian determinants. The deformation fields were then used to warp each parametric map (MT, PD, R1, R2*) to the common template. The resulting images were masked using the non-brainstem tissue class as an exclusion mask.

### Anatomical analysis

This work utilized two techniques to quantitatively analyze qMRI parameter maps. The first was TBM, which is a method to characterize volumetric change *in vivo*. It is similar to the already well-described voxel-based morphometry (Ashburner and Friston, 2000), however instead of statistically analyzing warped modulated tissue segmentations, the Jacobian determinant images are used (Ashburner and Friston, 2001). The second was VBQ. This is a pipeline to allow unbiased mass univariate statistical analysis of quantitative parameter maps whilst controlling for multiple comparisons with family-wise error (Draganski et al., 2011). The original VBQ method (Draganski et al., 2011) was modified slightly for this study. Because the brainstem lacks gyrification, and due to the highly accurate warping algorithm used, smoothing of the warped quantitative data was avoided, hence negating the need to produce “*warped-weighted average*” images that were required for the published approach. This had several advantages. First, only one statistical analysis was required for each quantitative map (i.e., the warped quantitative map) rather than one for each tissue class (i.e., four), which would have been necessary with the warped-weighted average approach. Additionally, because analysis was carried out directly on the warped quantitative maps without smoothing, a higher degree of spatial accuracy could be achieved.

### Statistical analysis

All statistical analysis was carried out using SPM8 in MATLAB 2010b. A two-sample *t*-test was initially used to check for scanner-associated differences in the acquisitions controlling for age, sex, and TIV, at FWE < 0.05 correction for multiple comparisons. No voxels survived correction. A design matrix for multiple linear regression model was then constructed including age, sex, and TIV as covariates. The TIV was centered around the mean, and the remaining covariates remained un-centered. An intercept was included in the model but no normalization was used. Using this design matrix, TBM was performed by analyzing the Jacobian determinant images (Ashburner and Friston, 2001), and VBQ by analyzing each warped quantitative map. For each analysis, the non-brainstem tissue class was used as an exclusion mask, ensuring only voxels that contained brainstem tissue were included. Each image type was assessed in a single design matrix to negate potential problems associated with uneven variances across the different quantitative maps. Each map was assessed for significant positive and negative correlates with age. For each contrast, FWE < 0.05 was reported. Finally, for each VBQ analysis where results were significant at FWE < 0.05, the T-maps were binarized for visualization at *p* < 0.001 uncorrected. These were used to create three-dimensional renderings, and also to assess the overlap of significant results between different modalities to better understand the interaction between the different measures. Finally, the same binarized images were used to extract the mean quantitative value for each individual. These values were plotted against the corresponding age, and the best linear fit indicated.

## Results

**Figure 3:**
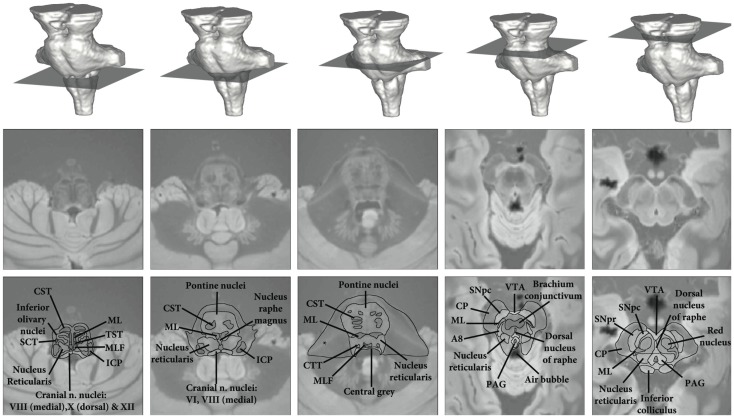
High resolution *ex vivo* combined MT T2* MRI with anatomical annotations for reference. Figure adapted from Lambert et al. (2013) with permission from Elsevier. Abbreviations: A8, dopaminergic center (approximate location), CP, cerebral peduncle (anterior to posterior: consisting of frontopontine, corticonuclear, corticospinal, and parietotemporal pontine tracts); CST, corticospinal tract; CTT, central tegmental tract; ICP, inferior cerebellar peduncle; ML, medial lemniscus; MLF, medial longitudinal fasciculus; PAG, periaqueductal gray; SCT, spinocerebellar tract; SNpc, substantia nigra pars compacta; SNpr, substantia nigra pars reticulata; TST, tectospinal tract; VTA, ventral tegmental area. *Artifact due to fixation.

For anatomical reference, a high resolution (0.5 mm isotropic) *ex vivo* combined MT T2* MRI with anatomical annotations has been provided in Figure 3 (figure adapted from Lambert et al., 2013 with permission from Elsevier).

### Tensor-based morphometry

**Figure 4:**
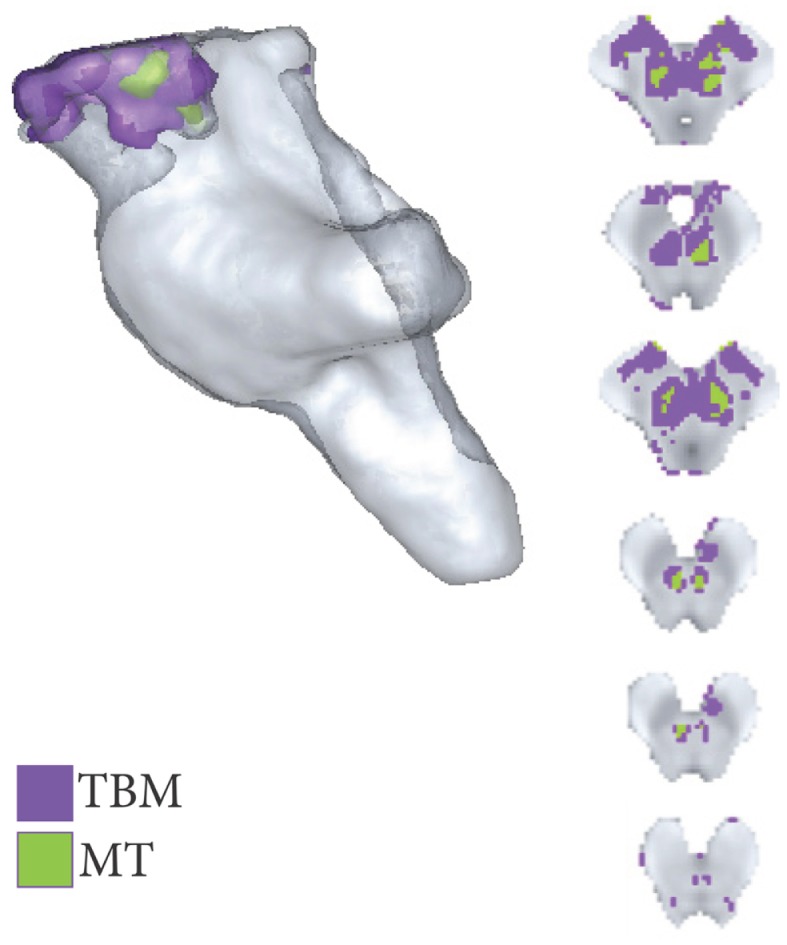
**Areas of significant regional decreases in tissue volume and magnetization transfer, binarized at *p* < 0.001 uncorrected**.

**Figure 5:**
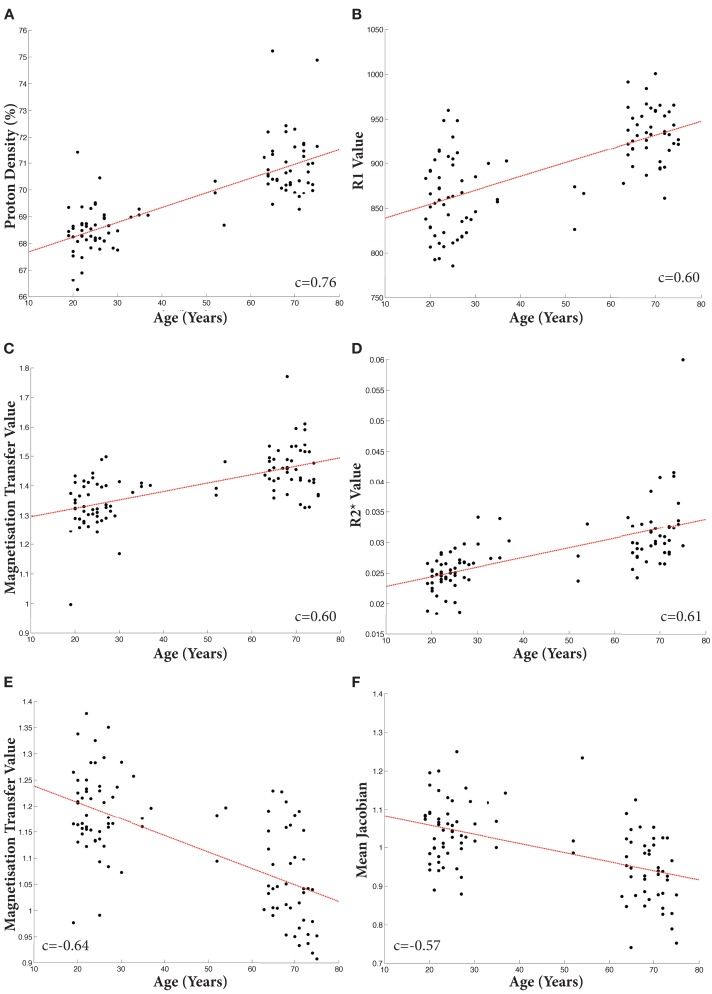
**Scatter plots of mean individual values extracted from binarized T-maps at *p* < 0.001 uncorrected**. Linear fit shown in red. Increases: **(A)** Proton Density; **(B)** R1; **(C)** Magnetization Transfer; **(D)** R2*. Decreases: **(E)**. Magnetization Transfer; **(F)**. TBM. All Pearson’s correlation coefficients significant at *p* < 1 × 10^−8^.

Highly localized volumetric decreases in tissue volume were found symmetrically within the brachium conjunctivum (superior cerebellar peduncle) bilaterally. These volumetric decreases were significant at FWE < 0.05 and are summarized in Figures 4 and 5. There were no significant positive TBM correlates with age at FWE < 0.05 and *p* < 0.001.

### Voxel-based quantification analysis

#### Negative correlates with age

Significant negative trends with age were only observed across the MT maps within the brachium conjunctivum at FWE < 0.05. These are summarized in Figures 4 and 5.

#### Positive correlates with age

**Figure 6:**
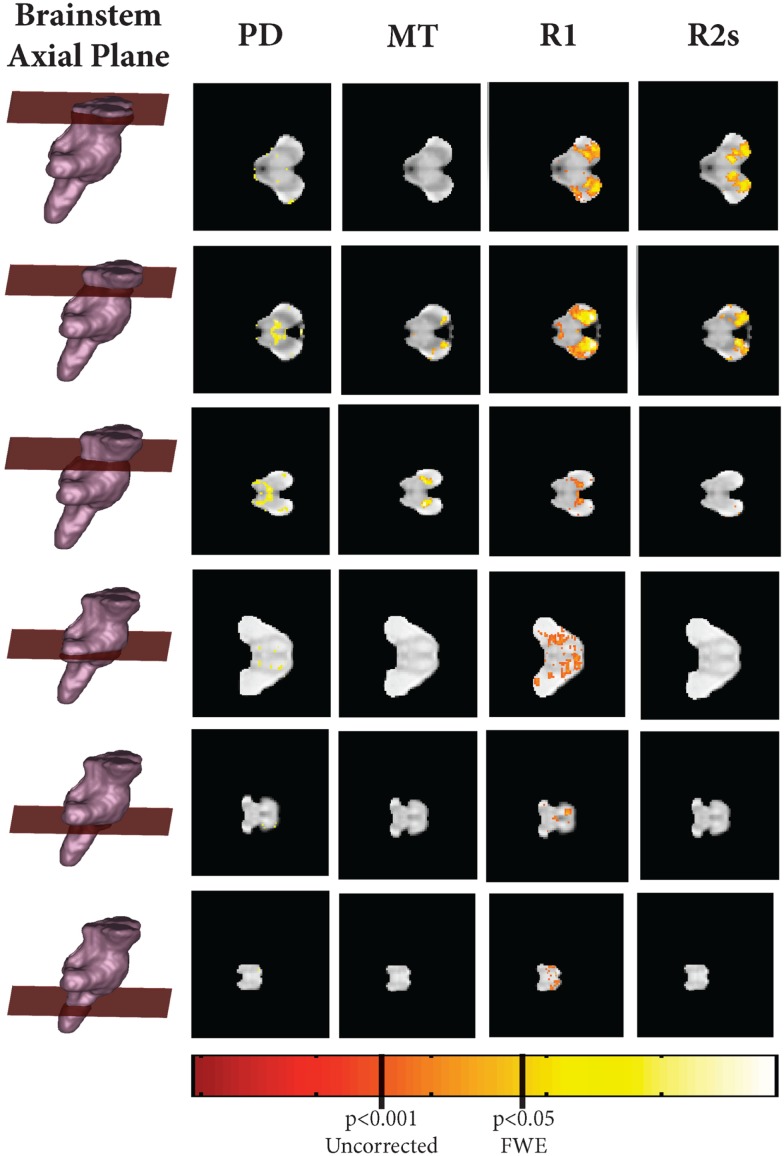
**Axial, coronal, and sagittal slice-wise images showing positive correlates with age (R2s, MT, A, R1) projected onto group-average MT map**.

**Figure 7:**
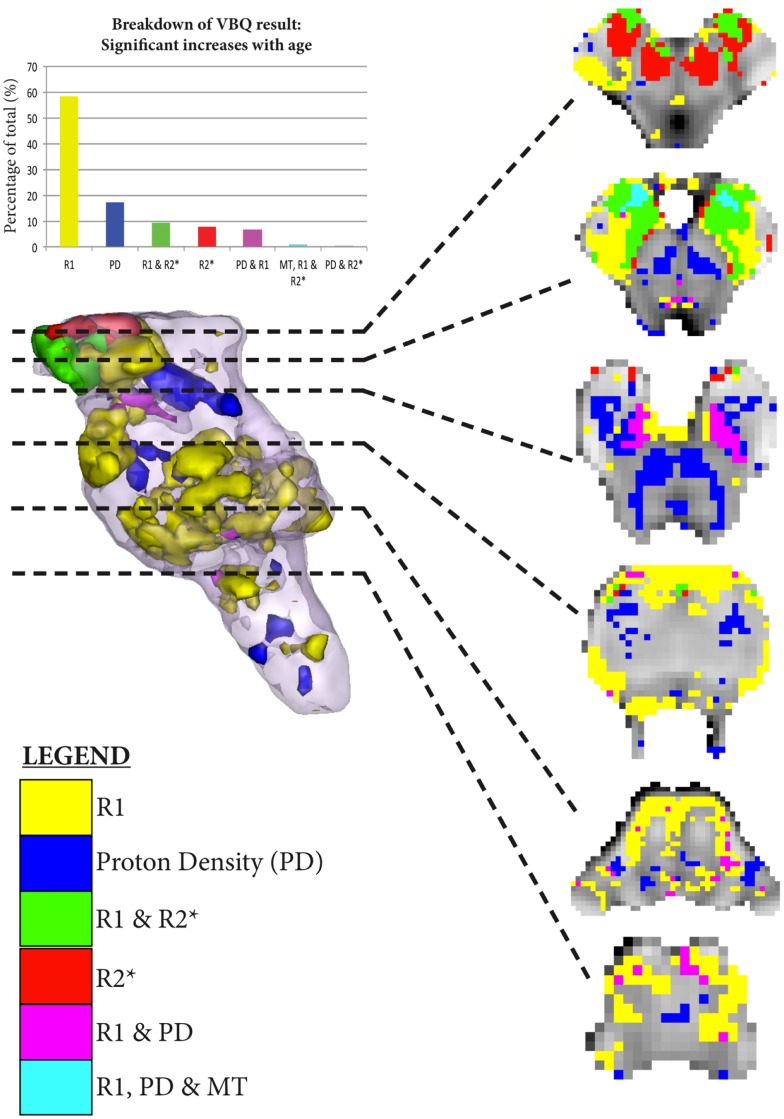
**Significant regional increases binarized at *p* < 0.001 uncorrected**. The legend classifies the regions of significant increase across the MPM, allowing overlapping significant increases to be simultaneously visualized. The histogram takes the total number of all significant voxels shown in the rendering, and then classifies them according to the MPMs in which those voxels are found in as per the legend.

As shown in Figures 5–7, there were more widespread significant increases in qMRI values with age. Figure 6 shows the individual parameter maps increases at six axial slices through the brainstem. Figure 7 summarizes the regional MPM increases and examines the overlap between the different parameter maps. It also demonstrates the relative proportions of each tissue type that overlap with one another.

##### R1 increases with age

Increases in R1 intensity values with age were found throughout the brainstem, but were confined to gray matter structures. These were found in bilateral inferior olivary nuclei, pontine nuclei, dorsal raphe nucleus, and bilateral substantia nigra. To better understand this finding, a sub analysis on these significant regions was performed, looking at the remaining parameters. Whilst there may be some selection bias with this approach (Vul et al., 2009), the correlation analysis was strictly performed to compare the behavior of the different MRI modalities within the regions of R1 change, to better understand what may be contributing to this R1 increase. A significant positive correlation with R2* was found within these regions, even when the iron rich midbrain structures were excluded (Pearson’s Correlation Coefficient within pons and medulla = 0.51, *p* < 1 × 10^−8^), implying that it is increasing iron deposition that significantly contributes to the observed R1 signal increases within brainstem gray matter structures.

##### R2* increases with age

Increases in R2* intensity values were localized to midbrain structures. Specifically, the substantia nigra, ventral tegmental area, and red nuclei. The pontine and medullary R1 regions described above did not show increases in R2s when analyzing the entire brainstem. This discrepancy would be due to correction for multiple comparisons at the whole brainstem level compared to a small volume region of interest.

##### Proton density increases with age

Significant increases in PD were observed in the brainstem white-matter. This included (from rostral to caudal): *Fasciculus* cerebellothalamicus, brachium conjunctivum, corticospinal tract, superior cerebellar peduncle, and medial longitudinal fasciculus. Additionally, the inferior portion of the substantia nigra also exhibited increased PD.

##### MT increases with age

Only one small region demonstrated MT increases: the corticobulbar portion of the cerebral peduncle bilaterally.

## Discussion

In this study, we have demonstrated the age effect of MT, R1, R2*, and PD in human brainstem using an automated, non-biased approach that is able to resolve the internal structure of the brainstem in unprecedented detail at 3T.

### Midbrain atrophy in healthy aging

Previous studies examining age-related volumetric decline in the brainstem have found no overall volume loss (Raz et al., 2001; Lee et al., 2009) but significant midbrain atrophy (Luft et al., 1999). This had previously been attributed to shrinkage of the substantia nigra (Raz, 1996), however there is sparse histopathological evidence to support this hypothesis (McCormack et al., 2004; Collier et al., 2007). Our study agrees with previous work in that brainstem aging-associated atrophy seems to be confined to the midbrain. However, our results indicate that it is predominantly volume loss of the superior cerebellar fiber bundles (brachium conjunctivum, fasciculus cerebellothalamicus (Haroian et al., 1981)] that are responsible for this finding. Additionally the decreasing myelin content, reflected by decreasing MT and increasing PD, indicates this is a regional effect due to axonal loss rather than an artifact. What is striking is the regional specificity of these findings. As with previous studies (Draganski et al., 2011), we found a marked increase in iron concentration with age in the substantia nigra and red nucleus. Though this increase is largely sequestered in neuromelanin *in vivo*, this substance will be released by dying neurons and hence can contribute to the regional damage observed (Chiueh, 2001; Papanikolaou and Pantopoulos, 2005). Not only is iron directly toxic to axons by causing rapid lipid peroxidation, it can also induce neurotoxic microglial factors to be released locally that will potentiate the regional insult (Zecca et al., 2004). These findings correlate with previously described cerebellar atrophy (Andersen et al., 2003; Draganski et al., 2011; Walhovd et al., 2011) and the resultant cortical and subcortical disconnection (Taniwaki et al., 2007; Alalade et al., 2011) hence would better account for these observations.

### Brainstem gray matter changes

Our results agree with previous studies in the observation that the pons and medulla do not show volumetric loss in aging (Sullivan et al., 2004; Lee et al., 2009). Despite this, we identified widespread and specific changes in the qMRI maps within the gray matter structures of these regions. Specifically, increasing R1 signal was observed within the pontine nuclei and inferior olive, in addition to the substantia nigra and dorsal raphe nuclei within the mesencephalon. This increase in R1 signal was significantly correlated with R2*, suggesting that increasing iron content within these structures can at least partly account for the observed gray matter changes. Within the pons, as with elsewhere in the brain, iron is predominately found in oligodendrocytes and to a lesser extent in the microglia and astrocytes (Ozawa et al., 1994). However the amount of iron in the former remains constant throughout aging and instead increases are found within the microglia and astrocytes (Zecca et al., 2004), which may relate to changes in vascular permeability. It has been speculated that the accumulation of immunoreactive iron in the microglia may cause or predispose to a neuroinflammatory response, as seen in Parkinson’s and Alzheimer’s disease (Zecca et al., 2004).

Chemical exchange brings a significant contribution to the transfer of magnetization between free water and macromolecules (Henkelman et al., 1993; Kucharczyk et al., 1994). Evidence has been presented which also suggests a significant impact of chemical exchange on the resonance frequency of water protons (Shmueli et al., 2011; Wharton and Bowtell, 2012). Physiological factors that impact chemical exchange such as tissue oxygenation (O_2_ and CO_2_ exchange), temperature, and pH (Kucharczyk et al., 1994; Liepinsh and Otting, 1996; van Zijl et al., 2003) may alter the MT and R2* estimates presented here. However only small variations of MT with pH have previously been reported within biologically plausible values (Kucharczyk et al., 1994). T1 relaxation is primarily driven by dipolar interactions between water and macromolecular protons (Koenig, 1995). Additionally the small variations in MT and T1 values of brain tissue with temperature (Lewa and Majewska, 1980; Graham et al., 1999) are unlikely to have a significant impact on our results within a range of biologically reasonable temperatures. Decreases in cerebral blood flow (CBF) that vary from region to region have been reported in normal aging (Aanerud et al., 2012). Modulated by the physiological parameters listed above, CBF may have an impact on the MPM measurements. However, the precise interaction between these remains poorly understood (Zauner and Muizelaar, 1997) and their impact on aging sparsely characterized. Several findings in this study suggest that the impact of CBF on healthy aging is minimal in the brainstem. First, biologically plausible concordance was accomplished between different parameter maps, for example the decreasing MT with increasing PD in areas of axonal and volume loss. Second, the results demonstrate respect for known anatomical boundaries such as the brachium conjunctivum with increased PD. Finally, our results are in agreement with previous histological observations, such as the increases in iron content in the substantia nigra (Zecca et al., 2001). Future work is expected to truly clarify and disambiguate the effects of CBF on MPM measurements. Not only will this improve the biophysical interpretation of these sequences in healthy tissue, but also allow better understanding of cerebral pathology where the vascular permeability will also change (Mooradian, 1988).

### Limitations

There are several limitations with our study. First, to accrue 100 normal control MPMs, we utilized scans that were acquired through previous studies (FitzGerald et al., 2012; Lambert et al., 2012; Chowdhury et al., 2013). There were few subjects between the ages of 35–65. Whilst this does not invalidate the findings, it makes it impossible to better characterize the temporal characteristics of the changes i.e., are they linear or non-linear. Additionally, as the upper age limit is 75, it is unclear how these changes extrapolate to those over that age. These features also bias the study toward those who are ambulatory, independent, and self motivating. Whilst it could also be argued that the latter criticism ensures that the experimental group consists only of healthy normal controls, it must also be acknowledged that declining mobility is a recognized feature of normal aging that may be unrelated to cortical changes, and this would certainly represent a selection bias in this work. Finally, it is currently unclear how these changes map to individual function, so further work is required to better understand this aspect.

In this work, the impact of physiological noise on image quality and the segmentation results was not explored. However, recent work using these techniques in the cortex highlights its robustness and also sensitivity to tissue microarchitecture (Dick et al., 2012; Sereno et al., 2013). Physiological noise has mostly been addressed in the context of fMRI, where image stability is paramount (Glover et al., 2000; Hutton et al., 2011). These methods cannot be directly implemented in anatomical imaging due to the different type of image acquisition. Potentially beneficial techniques include phase-navigator correction methods (Hu and Kim, 1994; Barry et al., 2008) although they may reduce the efficiency of the FLASH acquisitions. Alternatively real-time shimming methods for correction of respiratory-induced effects (Van Gelderen et al., 2007) or optical systems for fast prospective correction of subject motion (Zaitsev et al., 2006) may yield a significant reduction of physiological effects on anatomical scans.

Regarding the scanning parameters, 1 mm isotopic volumes are still reasonably large for certain brainstem structures, so our ability to fully characterize the changes are likewise limited. However, many structures in the brainstem are well above the 1 mm^3^ threshold such as the facial nerve nucleus (mean volume = 12.95 mm^3^) and hypoglossal nerve nucleus (mean volume = 14.39 mm^3^) (Sherwood et al., 2005), and hence this current study is of sufficient resolution for these nuclei. Additionally, we have demonstrated that widespread and structure specific changes can be found that also correspond to known features of brainstem aging.

### Future applications

This work provided a baseline of qMRI changes with aging in the brainstem. Further work is required to characterize the exact temporal dynamics of these changes, and how they extrapolate to those beyond the age of 75. Combining these techniques with diffusion-weighted imaging would allow further characterization of the changes within the white-matter and connectivity properties, and could be used to further refine the segmentations. Future work will examine these parameters in the context of the Parkinsonian disorders, such as idiopathic Parkinson’s disease, progressive supranuclear palsy, and multisystem atrophy. These conditions are good models of brainstem disease with which to develop the imaging techniques, as they all show post-mortem histological changes within the brainstem that, to date, are only evident on MRI at advanced stages of disease. Additionally, further longitudinal work is required to understand how individuals whose qMRI parameters lay well outside their expected normal age-matched distributions develop over time, to identify whether these parameters can serve as early biomarkers of neurodegenerative disease, an important prerequisite for any future neuroprotective therapy.

## Conclusion

In conclusion, we have characterized changes in the brainstem due to normal healthy aging using qMRI and volumetric analysis. We replicate previous findings of midbrain shrinkage, and put forward a new hypothesis as to the underlying mechanism based on statistically significant regional changes in the qMRI maps. Specifically, axonal loss in the ascending cerebellar fiber bundles is responsible for the decreased brainstem volume loss rather than atrophy of the substantia nigra, as previously speculated. Additionally, it may be that the regional increases in nigral and rubral iron content, as reflected by R2* signal, underpin these observations. Finally we demonstrate widespread brainstem changes during aging evidenced by increasing PD in the white-matter, and increasing R1 in the gray matter, the latter being significantly driven by increasing iron deposition. This work provides a baseline from which brainstem pathology can be better explored *in vivo* using 3T MRI, and non-invasive biomarkers of different neurodegenerative conditions.

## Conflict of Interest Statement

The authors declare that the research was conducted in the absence of any commercial or financial relationships that could be construed as a potential conflict of interest.
